# The Endogenous Dual Retinoid Receptor Agonist Alitretinoin Exhibits Immunoregulatory Functions on Antigen-Presenting Cells

**DOI:** 10.3390/ijms24119654

**Published:** 2023-06-02

**Authors:** Andreas Kislat, Peter Olah, Marcus Kuchner, Peter Arne Gerber, Jürgen Schrader, Stephan Meller, Bernhard Homey

**Affiliations:** 1 Department of Dermatology, Medical Faculty, University Hospital Düsseldorf, Heinrich-Heine-University Düsseldorf, 40225 Düsseldorf, Germany; 2 Department of Dermatology, Venereology and Oncodermatology, University of Pécs, 7622 Pécs, Hungary; 3 Institute for Molecular Cardiology, Medical Faculty, University Hospital Düsseldorf, Heinrich-Heine-University Düsseldorf, 40225 Düsseldorf, Germany

**Keywords:** alitretinoin, retinoids, chronic hand eczema, RAR, RXR, dendritic cells, human primary keratinocytes, 9-*cis* RA

## Abstract

Retinoids are a frequently used class of drugs in the treatment of inflammatory as well as malignant skin diseases. Retinoids have differential affinity for the retinoic acid receptor (RAR) and/or the retinoid X receptor (RXR). The endogenous dual RAR and RXR agonist alitretinoin (9-*cis* retinoic acid) demonstrated remarkable efficacy in the treatment of chronic hand eczema (CHE) patients; however, detailed information on the mechanisms of action remains elusive. Here, we used CHE as a model disease to unravel immunomodulatory pathways following retinoid receptor signaling. Transcriptome analyses of skin specimens from alitretinoin-responder CHE patients identified 231 significantly regulated genes. Bioinformatic analyses indicated keratinocytes as well as antigen presenting cells as cellular targets of alitretinoin. In keratinocytes, alitretinoin interfered with inflammation-associated barrier gene dysregulation as well as antimicrobial peptide induction while markedly inducing hyaluronan synthases without affecting hyaluronidase expression. In monocyte-derived dendritic cells, alitretinoin induced distinct morphological and phenotypic characteristics with low co-stimulatory molecule expression (CD80 and CD86), the increased secretion of IL-10 and the upregulation of the ecto-5′-nucleotidase CD73 mimicking immunomodulatory or tolerogenic dendritic cells. Indeed, alitretinoin-treated dendritic cells demonstrated a significantly reduced capacity to activate T cells in mixed leukocyte reactions. In a direct comparison, alitretinoin-mediated effects were significantly stronger than those observed for the RAR agonist acitretin. Moreover, longitudinal monitoring of alitretinoin-responder CHE patients could confirm in vitro findings. Taken together, we demonstrate that the dual RAR and RXR agonist alitretinoin targets epidermal dysregulation and demonstrates strong immunomodulatory effects on antigen presenting cell functions.

## 1. Introduction

In the early 20th century, vitamin A and its derivatives (retinoids) were discovered to be essential for mammalian health and survival [1]. The active form of vitamin A, retinol, is oxidized by alcohol dehydrogenases and retinal dehydrogenases in vivo to yield retinoic acid (RA) [2]. Retinol, the active form of vitamin A, is oxidized by alcohol dehydrogenases and retinal dehydrogenases in vivo to yield retinoic acid (RA) [2]. RAs exist in several isoforms but the predominant one is all-*trans* retinoic acid (ATRA) [3]. These naturally occurring RAs belong to the group of so-called retinoids, which also include synthetic compounds with structural or biological similarities to retinol [4]. Retinoids can be divided into three subgroups: 1st generation retinoids: natural or endogenous, including all*-trans* RA (ATRA), 9-*cis* RA (alitretinoin) and 13-*cis* RA (isotretinoin) [2]; 2nd generation retinoids: synthetic monoaromatic with acitretin and etretinate and 3rd generation retinoids: synthetic polyaromatic, including bexarotene, tazarotene and adapalene [5]. Retinoid signaling proceeds via binding to the ubiquitously expressed intracellular retinoic acid receptor (RAR) and/or retinoid X receptor (RXR) and thus mediates transcription or repression of downstream target genes [5,6]. While 13*-cis* RA (isotretinoin) as well as acitretin primarily bind to the RAR and bexarotene to the RXR, 9*-cis* RA (alitretinoin) shows affinity for both receptors and thus represents an endogenous dual retinoid receptor agonist [7]. Intracellular receptors as therapeutic targets are experiencing a renaissance in dermatology. Among these, retinoid receptors represent a well-known class, exerting effects not only on embryonic development, immunity and inflammation, but also on cell differentiation and wound healing [4,8,9,10]. Although their therapeutic use in numerous dermatoses, such as severe acne, psoriasis pustulosa, ichthyosis or cutaneous T-cell lymphoma, has proven successful [5,7,11,12], our understanding of the underlying mechanisms of action remains largely elusive. Chronic hand eczema (CHE) is a common inflammatory skin condition, occurring in up to 10% of the population and affecting the quality of life and work performance, especially in severe cases [13]. While topical therapeutics are the most common first-line option, a high percentage of cases require systemic treatment [14,15]. Since the endogenous dual retinoid receptor agonist 9*-cis* RA (alitretinoin) has been shown to be effective in treating severe CHE [16], we targeted this chronic inflammatory skin disease to obtain further insights into the role of RAR and RXR signaling in cutaneous homeostasis and inflammation.

## 2. Results

### 2.1. CHE as a Model Disease to Investigate the Effects of Alitretinoin (9*-cis* RA) on Skin Homeostasis and Inflammation

**Figure 1:**
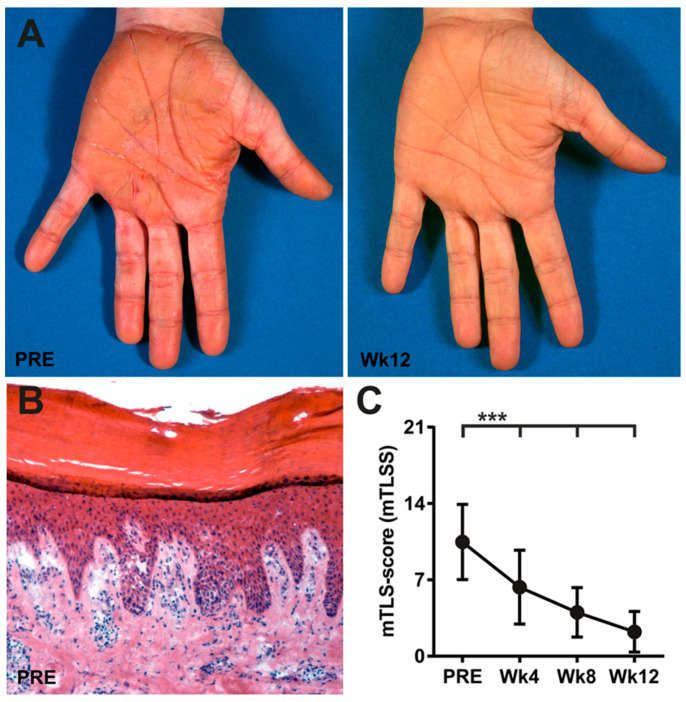
Treatment of CHE with alitretinoin (9*-cis* RA). (**A**) Representative clinical pictures of a 41-year-old patient with CHE before (PRE) and after 12 weeks (Wk12) of treatment with alitretinoin. (**B**) Representative dermatohistopathology of lesional palmar skin of a patient before treatment (Original magnification: 100×; hematoxylin and eosin). (**C**) Modified total lesion symptom score (mTLSS) of patients with CHE before and during treatment with alitretinoin (30 mg/d). Values represent the mean +/− SD of 23 patients (responders). Student’s *t*-test (*** *p* < 0.001).

In patients treated with alitretinoin, we used the modified Total Lesion Symptom Score (mTLSS) to assess the therapeutic efficacy on CHE. Twenty-three of twenty-nine CHE patients (79%) of the alitretinoin-treated patients showed a response of ≥50% in the mTLSS and were considered responders (Figure 1). Characteristics of the responder group are listed in supplementary Table S6. Among the selected alitretinoin-responders, a significant reduction of the mTLSS from 10.48 ± 3.37 before to 2.23 ± 1.82 after 12 weeks of treatment was observed (*p* < 0.001). This corresponded to an average reduction of the mTLSS by 78.63% (Figure 1).

### 2.2. Effects of Alitretinoin (9*-cis* RA) on Gene Expression in CHE and Cultured Human Primary Keratinocytes

**Figure 2:**
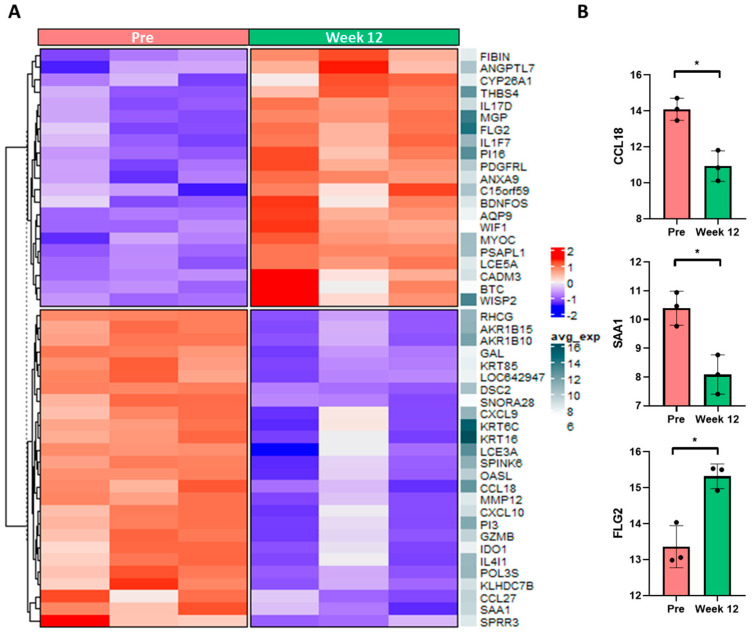
Significantly differentially expressed genes in skin biopsies of alitretinoin (9-*cis* RA)-treated patients. (**A**) Top 50 up- and downregulated genes between pre-treatment (top red bar, left column) and week 12 of treatment (top green bar, right column) as determined by cDNA microarray screening. Blue-red coloring indicates relative expression per individual gene (row-wise normalization), while the white-to-dark gray sidebar shows normalized mean expression across genes. (**B**) Boxplot of microarray gene expression values for selected pro-inflammatory genes and barrier components, indicating decreased inflammation and restoration of barrier function during treatment. Error bars: mean +/− SEM, black dots: individual samples. Generalized linear modeling of gene expression, adjusted *p*-value (* *p* < 0.05).

As a first step, we selected skin specimens of alitretinoin-responder CHE patients and performed transcriptome analyses. Using cDNA microarray analysis of skin biopsies of lesional CHE skin before and week 12 of alitretinoin treatment (n = 3), we detected a significant regulation of 231 genes with adjusted *p*-value < 0.05, log fold-change >1 in skin biopsies (Supplementary Table S1). Interestingly, gene expression patterns not only highlight down- and upregulated clusters (n = 95 and n = 136, respectively) but specific gene groups spanning a wide range of inflammatory, tissue remodeling, as well as barrier-related pathways. Downregulated genes include general inflammatory markers such as serum amyloid A1 (SAA1) and indoleamine 2,3-dioxygenase (IDO1) alongside chemokines CXCL9 and CXCL10 but also disease-specific markers such as CCL18 and CCL13. Levels of antimicrobial peptides (AMPs) typically upregulated during inflammation, such as peptidase inhibitor 3 (PI3/SKALP) or late cornified envelope genes LCE3A and LCE3C, are also normalized during treatment, similarly to matrix metalloproteinase 12 (MMP12). On the other hand, barrier function-related genes such as filaggrin 2 (FLG2), betacellulin (BTC) or Wnt inhibitory factor 1 (WIF1) are upregulated. Dermcidin, an AMP often suppressed in eczema, also shows a similar pattern (Figure 2, Supplementary Table S1). Interestingly, barrier function was not overrepresented among regulated genes, as compared to inflammatory processes or matrix remodeling. Functional enrichment analyses using two separate databases, Gene Ontology (GO) and the Bioplanet pathway resource, both point to significant enrichment in “extracellular matrix organization”, “IL1/TGF-beta regulation of extracellular matrix”, “Inhibition of matrix metalloproteinases” and “lymphocyte chemotaxis” (Supplementary Table S1). Furthermore, a functional network of top regulated genes was generated based on the expression and GO terms shared among genes, where distinct clusters highlight the above-mentioned regulated pathways (Supplementary Figure S4).

**Figure 3:**
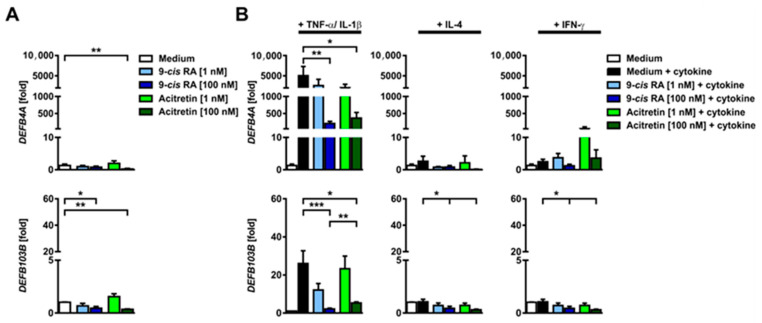
9-*cis* RA-treated keratinocytes show normalized expression of selected antimicrobial peptides following stimulation with TNF-α and IL-1β. Human primary keratinocytes were incubated either with or without alitretinoin (n = 7, independent donors) or acitretin (n = 4, independent donors) for 24 h and then harvested. Extracted RNA levels were measured by qPCR (normalized to 18S ribosomal RNA expression). (**A**) unstimulated controls. (**B**) human primary keratinocytes incubated either with or without alitretinoin (n = 7, independent donors) or acitretin (n = 4, independent donors) in the presence of TNF-α+IL-1β or IL-4 or IFN-γ for 24 h. Colored bars show mean + SEM of change compared with untreated controls without cytokine treatment. Mann–Whitney U test (* *p* < 0.05, ** *p* < 0.01, *** *p* < 0.001).

To gain further insights through the gene expression dataset, the FANTOM5 database was mined to extract cell types that are major sources of the above-mentioned genes (Supplementary Table S2). As expected, a remarkable number of the regulated genes are expressed in keratinocytes, identifying this cell subset as a major target for alitretinoin. In barrier defect-associated inflammation, IL-1β and TNF-α play a major role [17,18]. Thus, we simulated barrier defect-associated inflammation by stimulating cultured human primary keratinocytes with IL-1β and TNF-α in the presence or absence of alitretinoin or acitretin. The RAR agonist acitretin was selected as a comparator for the biological effects of the endogenous dual (RAR/RXR) agonist alitretinoin (9-*cis* RA). In addition, we also used key mediators of type 2 (IL-4) or type 1 (IFN-γ) inflammation to investigate the retinoid signaling driven effects on these conditions. A selection of genes based on both microarray data and previous findings was analyzed via qPCR, representing the main categories of AMPs, barrier function and tissue regeneration-related genes (Figure 3, Supplementary Figures S1–S3). Interestingly, under proinflammatory conditions with TNF-α and IL-1β, alitretinoin reduces the expression of top regulated genes small proline rich protein 3 (SPRR3) and serine protease inhibitor of Kazal type 6 (SPINK6). In turn, the opposite effect is observed at low doses of the sole RAR agonist acitretin. Furthermore, significant upregulation occurs in betacellulin (BTC) and hyaluronic acid synthase 2 (HAS2) under both retinoid treatments; however, the effect on HAS2 is found to be highly specific to alitretinoin across all conditions (Supplementary Figure S2). Regarding AMPs, defensin beta 4A and defensin beta 103B, which are often co-regulated with PI3 in inflammation, display extreme fold upregulation in stimulated keratinocytes under inflammatory conditions. However, even low doses of retinoids decrease the expression of these genes, and this effect is stronger with high dosages (Figure 3). On the other hand, RNASE7, S100A7 or hornerin (HRNR) do not show marked regulation under induced inflammatory conditions (Supplementary Figure S2). Drawing from the proinflammatory signature observed in vivo, a selected set of chemokines was also investigated in primary human keratinocytes under the above-mentioned conditions. The significant downregulation of CCL5, CCL20 and CXCL14 further supports the notion that 9-*cis* RA (alitretinoin) directly and indirectly regulates chemokine expression and thereby leukocyte recruitment (Supplementary Figure S5).

### 2.3. Morphology, Phenotype and Function of ‘Retinoid-DCs’ Generated from Isolated CD14^+^ Monocytes

**Figure 4:**
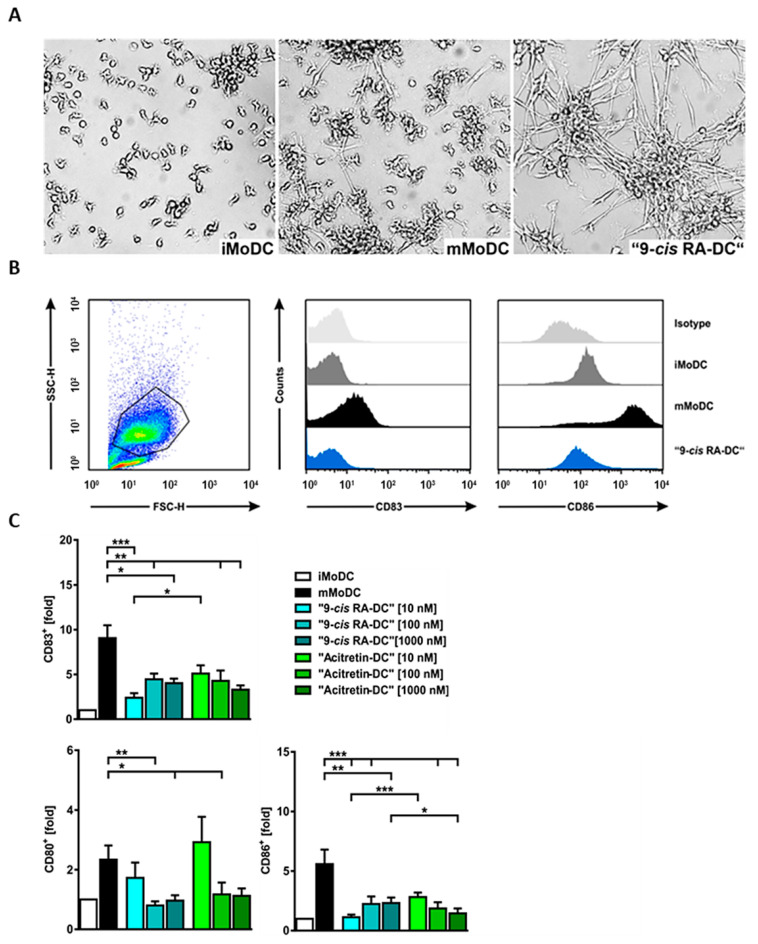
Morphological and maturation changes of 9-*cis* RA-treated monocyte-derived dendritic cells (MoDCs). (**A**) Representative microscopic images of immature monocyte-derived DC (iMoDC), maturated (mMoDC) and 9*-cis* RA [100 nM] treated TNF-a maturated cell (“9*-cis* RA-DC”) cultures after 9 days. Phase contrast light microscopy, magnification: 200×. (**B**) Representative half offset histograms of one donor show the expression of surface markers CD83 and CD86 and the corresponding isotype controls (light gray) on immature (iMoDC, dark gray), TNF-a maturated (mMoDC, black) and “9*-cis* RA-DCs” (blue) after 9 days of culture. 9*-cis* RA strongly downregulates CD83 and CD86 compared to mMoDCs. (**C**) Percentage of gated, positive cells for the maturation marker CD83 (n = 11–28 independent donors), co-stimulatory molecules CD80 (n = 11–12 independent donors) and CD86 (n = 11–25 independent donors). Fold changes are shown as mean + SEM of the percentage of gated cells positive for CD83, CD80 and CD86. Unpaired two-tailed Mann–Whitney U-test (* *p* < 0.05, ** *p* < 0.01, *** *p* < 0.001).

The FANTOM5 analysis also indicated that monocyte-derived cells are another prominent cell type that is targeted by alitretinoin (9-*cis* RA) (Supplementary Table S2). This is in good concordance with data previously published on all-trans retinoic acid (ATRA) [19,20,21]. Hence, we directed our focus on antigen presenting cells and dendritic cell functions. For this purpose, CD14^+^ monocytes were isolated from healthy donors and stimulated with GM-CSF and IL-4 to generate monocyte-derived dendritic cells (MoDC). After 6 days, these cells were matured with TNF-α (mMoDC) or left untreated to maintain an immature phenotype (iMoDC). These procedures were performed either with the cytokines alone or in the presence of 9-*cis* RA (alitretinoin) (‘9-*cis* RA-DCs’) or acitretin (‘Acitretin-DCs’). Upon treatment with 9-*cis* RA, a marked change in the morphology of dendritic cells was observed. Compared to untreated MoDC, ‘9-*cis* RA-DC’ exhibited an elongated shape with dense cell-to-cell connections (Figure 4).

**Figure 5:**
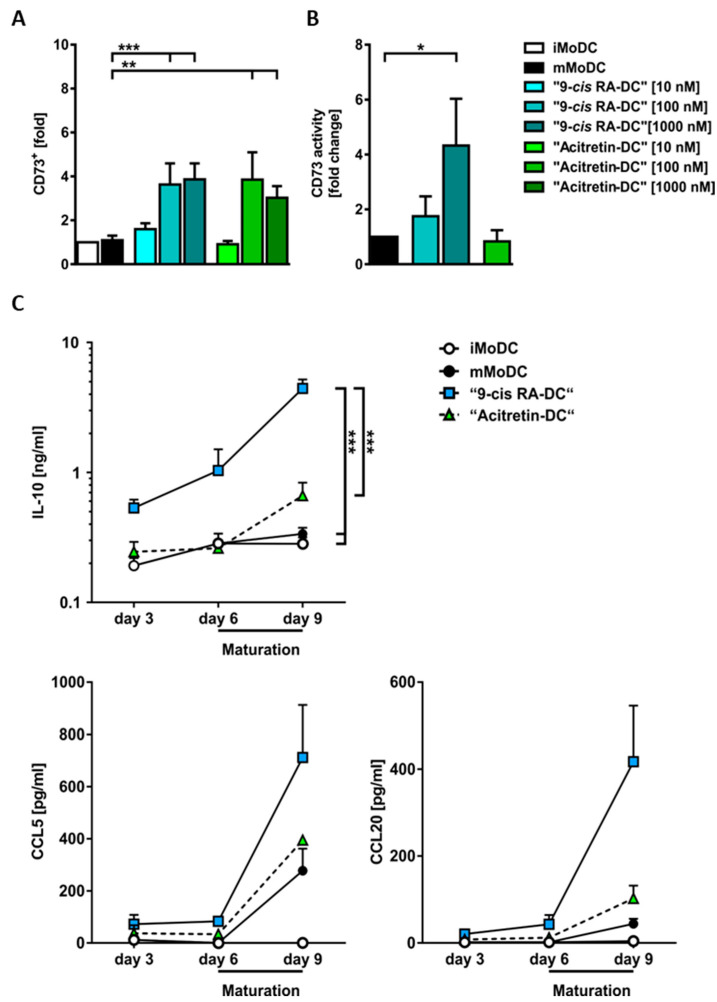
9-*cis* RA exerts immunoregulatory functions through inducing CD73 in dendritic cells and secretion of CCL5, IL-10 and CCL20 in the supernatants. (**A**) Expression of CD73 (n = 7–22 independent donors). Fold changes show mean + SEM of the percentage of gated, CD73-positive cells (**B**) CD73 enzyme activity of MoDCs. Data are expressed as a ‘‘fold change’’ considering mature MoDCs (mMoDC) of each individual donor (value = 1) (**C**) ELISA was performed to detect IL-10 (n = 6–12), CCL5 (n = 3) and CCL20 (n = 3). Mann–Whitney U test (* *p* < 0.05, ** *p* < 0.01, *** *p* < 0.001).

Given the profound morphological changes in ‘9-*cis* RA-DC’, we performed a transcriptomic screening comparing pooled RNA of six independent donors each of iMoDC, mMoDC and ‘9-*cis* RA DCs’ (Supplementary Figure S8, Supplementary Table S7). Considering the pooling strategy, only the top most robustly regulated genes were taken into account in downstream analysis. Of these, functional network clustering according to GO terms and fold regulation resulted in clusters enriched for: 1, antigen processing presentation (including CD80, CD83, TRAF and CTSD), 2, chemokine mediated signaling (CXCR5, CCL7 and CCL17) 3, negative regulation of cell proliferation (IL12B, OSM and EGR1) and 4, extracellular matrix regulation (including collagen and integrin family members as well as MMP12). Furthermore, qPCR analyses of a selected panel of cell type-relevant genes also confirmed 9-*cis* RA-specific transcriptomic changes in dendritic cells (Supplementary Figure S6). Since pathway analyses of transcriptomic changes induced by 9-*cis* RA in dendritic cells pointed towards antigen processing and presentation, we further investigated the phenotype of ‘9-*cis* RA-DC’ using flow cytometry. To assess the maturation status of ‘9-*cis* RA-DC’, we analyzed the surface expression of co-stimulatory molecules such as CD80, CD83 and CD86. Following maturation with TNF-α, an increased expression of CD80, CD83 and CD86 was observed (Figure 5, Supplementary Table S3). In contrast, cells matured in the presence of either 9-*cis* RA or acitretin showed decreased expression of these maturation markers. At equimolar doses, 9-*cis* RA was significantly more potent to maintain an immature phenotype and inhibit the cytokine-driven maturation process (Figure 5).

### 2.4. ‘9*-cis* RA-DC’ Show Functionally Active Expression of the Ecto-5’-Nucleotidase CD73 In Vitro

Immature dendritic cells are known to promote tolerance through the generation of regulatory T cells and a lack of T cell activation. Here, we show for the first time that retinoids upregulate the expression of the ecto-5′-nucleotidase CD73 in vitro and demonstrate that it is enzymatically active (Figure 5). CD73 produces adenosine from adenosine monophosphate, which can exert immunomodulatory functions on T cells via adenosine receptors, suggesting a possible mechanism of action of 9-*cis* RA [22,23,24,25,26]. Maturation had no effect on mMoDCs as opposed to iMoDCs. The addition of 100 and 1000 nM 9-*cis* RA or 100 nM acitretin significantly upregulated the expression of CD73 (n = 7–22 independent donors) on the cell surface of MoDCs (Figure 5). To test the enzyme activity of CD73, etheno-AMP degradation to etheno-adenosine was measured. CD73 was not only expressed on the cell surface but also active with enzymatic activity increasing with addition of 100 nM 9-*cis* RA by 1.75-fold and with 1000 nM 9*-cis* RA (*p* = 0.0286) by 4.3-fold compared to mMoDCs (Figure 5). Using a cytokine array, we also examined the expression of various cytokines and chemokines in the cell supernatant (Supplementary Figure S7) and verified this by ELISA for selected cytokines. After stimulation with alitretinoin, dendritic cells showed a marked secretion of IL-10, an immunoinhibitory cytokine, and an increased secretion of CCL5 and CCL20 was shown. The effect of alitretinoin is more pronounced than for acitretin (Figure 5).

### 2.5. Reduction of Proliferative Response of Allogenic Stimulated CD3^+^ T Cells by Retinoids 

**Figure 6:**
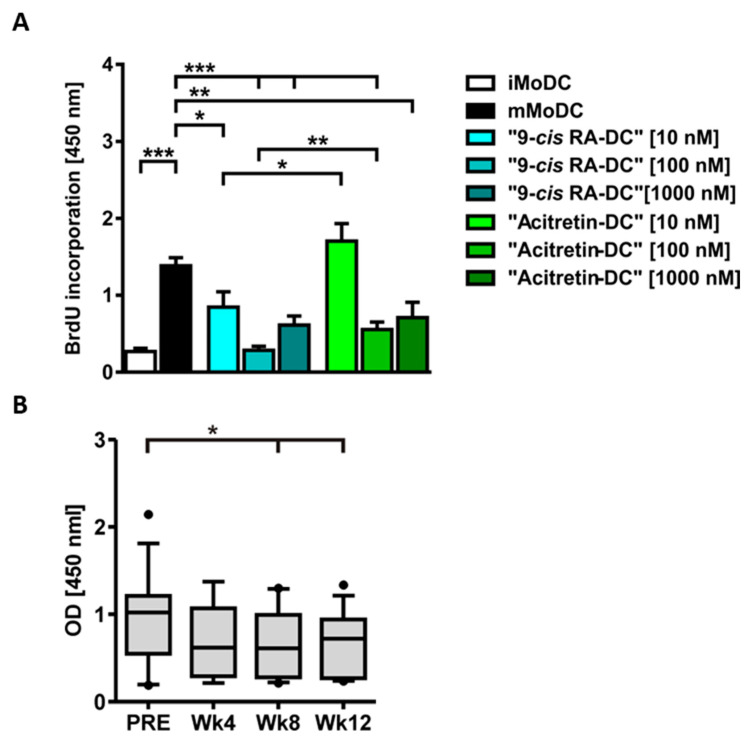
9-*cis* RA induced a marked change on the phenotype of CD14^+^ monocytes and exerts effects on the capacity of PBMCs to stimulate T cells. (**A**) Detection of proliferation of CD3^+^ T cells by BrdU incorporation after allogeneic stimulation with MoDCs. Allogenic stimulation was performed with iMoDCs, mMoDCs and retinoid DCs. Retinoid DCs were treated with different concentrations of alitretinoin and acitretin. Cells were then incubated with allogeneic T cells. Detection was performed after 6–7 days. The MoDC: T cell ratio was 1:200. Results are expressed as mean + SEM, data constitute optical density (OD) of 6–28 independent donors. Means differed significantly by unpaired two-tailed Mann–Whitney U test (* *p* < 0.05, ** *p* < 0.01, *** *p* < 0.001). (**B**) PBMCs of 9-*cis* RA-treated CHE patients were co-cultivated with allogenic T cells from healthy donors. Graph shows BrdU-incorporation as extinction coefficient [450 nm] as box and whiskers. The whisker lines indicate 10% and 90% values; the black vertical lines represent median values; the outliers are represented as filled circles. The means were significantly different for week eight (*p* = 0.0285) and week twelve (*p* = 0.0346) compared to baseline proliferation using a one-tailed paired Student’s *t*-test (* *p* < 0.05).

In view of the altered maturation markers, the higher expression of CD73 as well as the release of IL-10, the question arose whether retinoid-treated dendritic cells have an immunomodulatory effect on other cell types such as T cells. iMoDCs, mMoDCs and concentration-dependent matured “Alitretinoin- and Acitretin-DCs” were co-cultured with allogenic T cells at a ratio of 1:200. Proliferation was measured by BrdU incorporation after 6–7 days (Figure 6). Interestingly, after maturation in the presence of retinoids, DCs showed a significantly lower potential to lead to T cell proliferation, which also underlines the immunomodulatory functional significance of retinoids. In equimolar doses, alitretinoin is significantly more effective than acitretin (Figure 6). To confirm the inhibitory effect on T cell proliferation of 9-*cis* RA treated immune cells in vivo, PBMCs from patients suffering from CHE before and after week four, eight and twelve of treatment with 9-*cis* RA were isolated and cultivated with allogenic T cells isolated from healthy donors. At day six, the proliferation rate of leukocytes was measured by BrdU-incorporation. BrdU incorporation was analyzed with one-tailed paired Student’s *t*-test. 9-*cis* RA treatment significantly decreased T cell proliferation following allogenic stimulation after week eight (*p* = 0.0285) and week twelve (*p* = 0.0346) when compared with T cell proliferation rates before the treatment (Figure 6). In addition, the effect of alitretinoin treatment was assessed on the phenotype of CD14^+^ monocytes in patient samples, where the percentage of CD14^+^CD86^+^ monocytes was significantly reduced after 12 weeks of treatment with oral 9-*cis* to baseline (*p* = 0.027). Moreover, CD14^+^CD73^+^ cells were upregulated during treatment at week four (*p* = 0.0043) and week twelve (*p* = 0.016) (Supplementary Figure S9).

## 3. Discussion

Here, we present a detailed characterization of immunomodulatory effects of the dual retinoid receptor agonist alitretinoin in the context of CHE. Microarray data collected during the course of 12-week treatment provided a wide perspective on the potential mechanisms and affected pathways, highlighting targets for downstream in vitro analyses. Although the effects of retinoids on tissue remodeling pathways and metalloproteinases are well established, among the top regulated genes we also find hallmark chemokines CCL18 and CCL13, alongside inflammatory markers SAA1 or IDO1. The normalization in expression levels of these genes by week 12 is a strong indicator of reduced inflammation, while also outlining closely co-regulated genes with less clear roles in the pathology. Notably, AMPs such as PI3 or LCE3A are also downregulated, while DCD, an antimicrobial component of sweat typically downregulated in eczema [27], shows the opposite direction. Members of the defensin family were not found among significantly regulated genes in vivo; however, follow-up experiments on stimulated keratinocytes showed very high upregulation of DEFB4A and DEFB103A, both well-known players in inflammatory skin conditions, with remarkable downregulation upon alitretinoin treatment. Similar patterns were observed for RNASE7 but not hornerin (HRNR), while DCD was even upregulated upon alitretinoin treatment. This suggests that homeostatic levels of antimicrobial activity are restored, which aligns well with the clinical observation that alitretinoin treated patients do not present a significantly increased frequency of infections. Regarding skin structure and barrier constituents, SPRR family genes also show high levels of regulation and are increasingly investigated in skin disease [28,29]. Here, the eczema-associated gene SPRR3 shows prominent fold regulation, indicating barrier function-related alterations alongside FLG2, AQP9 and several keratins in vivo. On the other hand, investigating the expression of key hyaluronan synthase and hyaluronidase genes, alongside betacellulin, significant upregulation of synthases, particularly HAS2, is observed under alitretinoin treatment. Hyaluronidase genes are however not affected by alitretinoin, suggesting a positive effect towards matrix remodeling and cutaneous regeneration. Moreover, a decrease in the amount of hyaluronan fragments due to the imbalance of hyaluronan production and lower degradation may also lead to impaired inflammatory responses and a thickening of the epidermis, as previously observed [30]. 

In the analysis of significantly regulated genes using the FANTOM5 database, antigen presenting cells were identified to be the main cellular targets of alitretinoin, besides keratinocytes. The presented findings indicate that dendritic cells treated with 9-*cis* RA (alitretinoin) remain in an immature state with low co-stimulatory molecule expression (CD80 and CD86). The mMoDC phenotype presented here resembles the “TNF-α/CD40L” phenotype with an upregulation of key indicator genes previously described [31,32]. ‘9-*cis* RA-DCs’ demonstrated a decreased expression of maturation-associated genes CD83, CD80, CD40, CCL17 and CCL22 in comparison to mMoDCs. Markers such as CD14 or CD163 were upregulated compared to mMoDCs. Altogether, on the transcriptional level, “9-*cis* RA-DCs” demonstrated a more iMoDC-like expression pattern and supported the hypothesis that RA-treatment shapes monocytes/DCs into immunomodulatory DCs, which have the ability to down-modulate T cell responses and control inflammatory processes. These observations were further supported by results obtained by flow cytometry of MoDCs which showed that 9-*cis* RA (alitretinoin) as well as acitretin have an inhibitory effect on the differentiation and maturation of DCs. An inhibitory effect on the maturation and differentiation with ATRA has been previously observed (1 µM and 10 nM). CD80 and CD86 were downregulated in both concentrations upon GM-CSF + IL-4 stimulation and additional TNF-α maturing [31]. Additionally, ATRA reduced the percentage of CD83^+^ HLA-DR^+^ mature dendritic cells [32,33,34]. Overall, these results confirmed findings on other RAs and showed that 9-*cis* RA inhibited the maturation of dendritic cells by reducing co-stimulatory molecules CD80 and CD86. Impairment of CD86 and CD80 expression on macrophages and dendritic cells leads to a reduced activation and a lower cytokine production of T cells [35,36]. As a result of 9-*cis* RA (alitretinoin), ‘retinoid-DCs’ have a lower activation potential and might function as immunomodulatory cells. Further, the endogenous retinoid alitretinoin (9-*cis* RA) is not only effective in reducing maturation of TNF-α stimulated DCs in vitro, but it is more effective than the synthetic RAR agonist acitretin. To evaluate whether soluble factors may also support immunoregulation, we performed a cytokine array of the supernatants of mMoDCs and ‘9-*cis* RA-DCs’. We observed a significantly increased secretion of the anti-inflammatory cytokine IL-10 in the supernatants of ‘9-*cis* RA-DCs’. These results were confirmed by ELISA showing a higher efficacy to release IL-10 in response to alitretinoin when compared with equimolar doses of acitretin. Hence, transcriptomic, flow cytometric and secretory analyses pointed towards an immunomodulatory phenotype of 9-*cis* RA-DC. To further explore this notion, we investigated the adenosinergic pathway. ENTPD1 (CD39) and the downstream CD73 break down adenosine triphosphate into adenosine monophosphate and finally into adenosine [37,38]. Enzyme activity was tested, demonstrating not only the presence of CD73 but also the increased activity of the molecule on the surface of ‘9-*cis* RA-DCs’. Findings of the present study demonstrate for the first time that 9-*cis* RA and acitretin can induce CD73 on the surface of human moDCs and that the molecule is functionally active. These findings support the role of CD73 in the regulation of immune functions of DCs, and findings indicate a new role for retinoid receptor signaling by inducing CD73 and resulting in an immunomodulatory response [39,40,41]. To show the direct impact of ‘9-*cis* RA-DC’, we demonstrated a significantly reduced allogeneic T-cell response in vitro and confirmed this inhibitory effect on T cell activation and proliferation using PBMCs of alitretinoin-treated CHE patient ex vivo. Hence, these findings strongly support that alitretinoin (9-*cis* RA) is inducing an immunomodulatory phenotype in dendritic cells in vitro and in vivo. Taken together, we demonstrate that the dual RAR and RXR agonist alitretinoin targets epidermal dysregulation and demonstrates strong immunomodulatory effects on antigen presenting cell functions. 

## 4. Materials and Methods

### 4.1. Cell Lines and Reagents

Skin samples, serum samples and PBMCs were collected from patients with CHE (n = 29) as part of routine diagnostic testing. Informed consent was obtained for further processing of the respective samples. Patients received 30 mg 9-*cis* RA orally, daily for 12 weeks. One of 29 patients received 10 mg 9-*cis* RA in the first four weeks of treatment. Human primary keratinocytes, isolated from healthy donors (breast reduction or foreskin), were cultured in keratinocyte medium (GIBCO, Invitrogen, Carlsbad, CA, USA) containing recombinant EGF (0.1–0. 2 ng/mL), bovine pituitary extract (20–30 μg/mL), L-glutamate (2 mM, PAA, Pasching, Austria), 1% penicillin (100 U/mL) and streptomycin (100 μg/mL) (PAA, Pasching, Austria). Human primary monocytes/dendritic cells were cultured in RPMI 1640 GlutamMax (GIBCO, Invitrogen, Carlsbad, CA, USA) with 10% fetal calf serum (FCS) (Biochrome AG, Berlin, Germany) and 1% penicillin 100 U/mL, streptomycin 100 μg/mL) (PAA, Pasching, Austria). Stimulation was performed with GM-CSF (100 ng/mL) (GenScript, Piscataway, NJ, USA) and interleukin (IL-4) (50 ng/mL) (R&D Systems, Minneapolis, MN, USA). Cells were incubated at 37 °C with 95% humidity and 5% CO_2_ (INCO 2, Memmert, Schwalbach, Germany). Cytokines were reconstituted in 1% BSA/PBS to a final concentration of 100 µg/mL. Alitretinoin stock solution (100 mM) (Basilea Pharmaceuticals, Basel, Switzerland) and acitretin stock solution (10 mM) (Basilea Pharmaceuticals, Basel, Switzerland) were diluted in DMSO (Sigma-Aldrich, Saint Louis, MO, USA). Keratinocytes were pre-treated for 48 h in the presence or absence of alitretinoin or acitretin (1, 100 nM). Subsequently, cells were treated with rhTNF-α (10 ng/mL) (AbD Serotec, Oxford, UK) plus rhIL-1β (5 ng/mL) (R&D Systems, Minneapolis, MN, USA), rhIL-4 (50 ng/mL) (R&D Systems, Minneapolis, MN, USA) or rh Interferon-γ (IFN-γ) (10 ng/mL) (R&D Systems, Minneapolis, MN, USA) or medium mixed in the presence or absence of alitretinoin (9-*cis* retinoic acid) or acitretin (1, 100 nM).

### 4.2. Isolation of Peripheral Blood Mononuclear Cells (PBMCs), Monocytes and Generation of Monocyte-Derived Dendritic Cells (MoDCs)

PBMCs were isolated from peripheral blood samples using BD Vacutainer^®^ CPT™ Cell Preparation Tube with Sodium Citrate (BD Biosciences, San Jose, CA, USA) according to the manufacturer’s protocol. Buffy coats were obtained from the Institute of Hemostasis and Transfusion Medicine, University Hospital, Düsseldorf, Germany. The buffy coat cell suspension was diluted 1:2 with RPMI 1640 (GIBCO, Invitrogen, Carlsbad, CA, USA). A total 37.5 mL of cell suspension was layered on top of 12.5 mL Ficoll-Paque Plus^TM^ solution (GE Healthcare, Buckinghamshire, UK) and was separated by centrifugation (1150 rpm, 20 min, room temperature (RT)) (Rotina 46 R, Hettich, Bäch, Switzerland) without break. The interphase was carefully transferred and washed in RPMI 1640 (GIBCO, Invitrogen, Carlsbad, CA) and centrifuged. Erythrocytes in the pellet were lysed by ammonium chlorid lysis buffer and incubated at 4 °C for 10 min. Remaining cells were washed in PBS. CD14-positive monocytes were isolated by magnet beads (R&D Systems, Minneapolis, MN, USA) according to the manufacturer’s protocol. Monocyte separation was performed by using the MagCellect Magnet (R&D Systems, Minneapolis, MN, USA). To generate MoDCs, CD14-positive monocytes were cultured in RPMI 1640 (Invitrogen, Carlsbad, CA, USA), 10% fetal calf serum (FCS) (Biochrome AG, Berlin, Germany) and penicillin/streptavidin (PAA, Paching, Austria) and stimulated with rhGM-CSF (100 ng/mL) (GenScript, Piscataway, NJ, USA) and rhIL-4 (50 ng/mL) in the presence or absence of alitretinoin or acitretin (10, 100 or 1000 nM). Medium change was performed after 3 days. At day 6, a half of the medium was replaced with rhGM-CSF (100 ng/mL) and rhIL-4 (50 ng/mL) and rhTNF-α (100 ng/mL) in the presence or absence of alitretinoin or acitretin (10, 100 or 1000 nM). Dendritic cells were harvested at day 9.

### 4.3. Flow Cytometry Analysis

The lymphocyte subsets were analyzed by using specific antibodies and FACS Calibur flow cytometer (BD Biosciences, San Jose, CA, USA). At least 50,000 events were counted in every tube. The specific antibodies are listed in Supplementary Table S4. Analysis was done by FACScan and CellQuest v5.2 software (Becton Dickinson, Mountain View, CA, USA) or FlowJo (FLOWJO, LLC, Ashland, OR, USA) and visualized in a histogram plot (counts over fluorescence).

### 4.4. RNA Extraction

RNA extraction of the cells was performed using TRIzol^®^ reagent (Invitrogen, Carlsbad, CA, USA) and addition of 0.2 volume chloroform (Merck, Darmstadt, Germany). The cell suspension was vortexed and centrifuged (15 min, 12,000 rpm, 4 °C), and the supernatant was transferred to a new tube. RNA precipitation was performed using 0.5 volume isopropanol (Merck, Darmstadt, Germany). This was followed by incubation at −20 °C overnight. The supernatant was discarded. The RNA pellet was washed in 80% ethanol (Merck, Darmstadt, Germany) and centrifuged (12,000 rpm, 30 min, 4 °C) and dissolved in H_2_O. The RNeasy Mini Kit (QIAgen, Hilden, Germany) was used for RNA extraction from the tissue samples according to the manufacturer’s protocol.

### 4.5. cDNA Microarray Analysis

Extracted RNA was hybridized to Agilent G3 Human Gene Expression Microarrays (G4851C) according to manufacturer protocols. Quality metrics were generated using ArrayQualityMetrics. Differential gene expression analyses were conducted on RMA-normalized probe intensity values summarized at the gene level in the R package limma, considering as cutoffs absolute log-fold change >0.5 and adjusted *p*-value < 0.05, with further narrowing down of gene lists for visualization. Functional enrichment analysis has been carried out using Panther v15. Functional gene networks were visualized in Cytoscape v3.6.

### 4.6. Quantitative Real Time (qPCR) Analysis

For qPCR, 18S RNA gene expression was used as an internal standard. Quantitative PCR was performed using the Applied Biosystems 7000 System/Quantstudio 6 and Power SYBR^®^ Green PCR Master Mix or TaqMan^®^ Universal PCR Master Mix (Applied Biosystems Inc., Foster City, CA, USA) according to the manufacturer’s instructions.

### 4.7. Primer Design

Oligonucleotides for qPCR were either designed on mRNA sequences deduced from GenBank or obtained as TaqMan^®^ Gene Expression Assays by Applied Biosystems (Supplementary Table S5).

### 4.8. ELISA

Cytokine and chemokine concentrations in the supernatants were measured by enzyme-linked immunosorbent assay (ELISA DuoSet, R&D Systems, Minneapolis, MN, USA) according to the manufacturer’s protocols. The optical density of each well was determined using an ELISA reader (Multiscan Ascent, Thermo Fisher Scientific, Wilmington, DE, USA). Sample concentrations were calculated against standard curves and with the Graphpad Prism software (version 5.03, GraphPad Software, La Jolla, CA, USA).

### 4.9. Cytokine Array

We analyzed the supernatants of monocytic dendritic cells matured with TNF-α with or without alitretinoin [100 nM] and examined them with the Human Cytokine Array C5 (Ray Biotech, Norcross, GA, USA) according to the manufacturer’s protocol. We performed the calculations using ImageJ software v1.42 (National Institutes of Health, Bethesda, MD, USA) against internal standards as a percentage of positive control. We considered a fold log_2_ difference of more than 1.5 to be relevant.

### 4.10. Isolation of T Cells

Isolated PBMCs were resuspended in RPMI 1640. Depletion of monocytes and macrophages was carried out in 175 cm^2^ cell culture flasks (Greiner Bio-One GmbH, Kremsmünster, Austria) at standard cell culture conditions as monocytes and macrophages will adhere to the wall of the plastic flask. Therefore, the non-adherent cells were suspended in 20 mL of RPMI 1640, centrifugated as above and resuspended in 4 mL of 1 × column wash buffer (R&D Systems, Inc., Minneapolis, MN, USA). T cell isolation was performed by the use of human T cell enrichment columns (R&D Systems, Inc., Minneapolis, MN, USA) according to the manufacturer’s instructions. Finally, T cells were resuspended in appropriate medium.

### 4.11. Mixed Leukocyte Reaction

The influence of 9-*cis* retinoic acid and acitretin on allogenic stimulation of monocyte-derived dendritic cells was analyzed. Briefly, 5 × 10^3^ monocyte-derived immature and mature dendritic cells (DC) pre-treated in the presence or absence of 9-*cis* RA or acitretin (10, 100 or 1000 nM) and 1 × 10^5^ purified T cells were co-cultured for 6–7 days in 96-well roundbottom-plates in X-Vivo 15 media (Lonza, Basel, Switzerland). Proliferation of leukocytes was measured by BrdU-incorporation (Roche, Basel, Switzerland) after 6–7 days in a 96-well plate reader (Multiskan Ascent, Thermo Fisher Scientific, Wilmington, DE, USA). Some 1 × 10^5^ isolated Patient-PBMCs before and 12 weeks after initiation of 9-*cis* RA treatment were co-cultured with 1 × 10^5^ enriched T cells for 6 days. Proliferation was measured by BrdU-incorporation after 6 days in a 96-well plate reader.

### 4.12. CD73 Enzymatic Activity

Dendritic cells (5 × 10^4^) were incubated in RPMI 1640 GlutaMaxII (Invitrogen, Carlsbad, CA, USA), 10% fetal calf serum (FCS) (Biochrome AG, Berlin, Germany) and penicillin/streptavidin (PAA, Paching, Austria) and incubated as described previously [42]. Cells were incubated for 1h with or without 50 µM AOPCP (Tocris, Bristol, UK). The reaction was started with 50 µM Etheno-AMP (Biolog, Bremen, Germany). After 10, 20, 30, 40, 50 and 60 min, 15 µL of the supernatant was taken. The reaction was stopped by mixing the super-natant with the same volume 1 M perchloric acid and stored at −20 °C until further analysis. For HPLC analysis, samples were neutralized with 1 M K_3_PO_4_. Etheno-AMP, and etheno-adenosine amounts were quantified using a 1525 Binary HPLC pump (Waters GmbH, Eschborn, Germany). Separation of the two analytes was accomplished on an XTerra MSC18 Column (Waters GmbH, Eschborn, Germany). Solution A contained 6% (*v*/*v*) acetonitrile/5.7 mM tetrabutylammonium bisulfate/30.5 mM KH_2_PO_4_, pH 5.8. Solution B consisted of 66% (*v*/*v*) acetonitrile/5.7 mM tetrabutylammonium bisulfate/30.5 mM KH_2_PO_4_, pH 5.8. Areas from the appropriate chromatograms were used for determination of reaction rate which is given as enzymatic activity per 1 × 10^6^ cells by using Waters Breeze software v1.4 (Waters GmbH, Eschborn, Germany). Samples were measured in triplicate.

### 4.13. RNA Sequencing

RNA preparations were checked for RNA integrity by Agilent 2100 Bioanalyzer quality control. Data quality was screened and filtered using FastQC v0.11.9 (Babraham Bioinformatics group, Cambridge, UK) and the fastx toolkit v0.0.14 (Hannon Lab—Cold Spring Harbor Laboratory, Cold Spring Harbor, NY, USA), followed by DESeq2 v3.17 analysis [43]. Reduction of spurious fold change estimations due to moderate sample size was achieved by discarding genes exhibiting the lowest 5% of expression, log2 fold-change <1 and the false discovery rate > 0.05.

### 4.14. Statistics

Data were expressed as mean +/− standard deviation (SD) or +/− standard error of the mean (SEM). Paired or unpaired Student’s *t*-test or Mann–Whitney-U-test was performed using Graphpad Prism software (version 5.03, GraphPad Software, La Jolla, CA, USA). (* *p* ≤ 0.05, ** *p* ≤ 0.01, *** *p* ≤ 0.001). Heatmaps and network figures were generated in R v.3.5 and Cytoscape v3.6. Methodologies were adapted from the dissertation of author Dr. Andreas Kislat, the source material of the present publication [44].

## Data Availability

Raw data files of all measurements performed in the study are available from the corresponding author upon reasonable request.

## Supplementary Materials

The supporting information can be downloaded at: https://www.mdpi.com/article/10.3390/ijms24119654/s1
