# Supplementary material for: The Endogenous Dual Retinoid Receptor Agonist Alitretinoin Exhibits Immunoregulatory Functions on Antigen-Presenting Cells
Source: Int J Mol Sci. 2023 Jun 2;24(11):9654. doi: 10.3390/ijms24119654 (PMC10253969; doi:10.3390/ijms24119654)
Supplement: Supplementary file 1 [file ijms-24-09654-s001.zip › Kislat_2023_Supplementary_Figures_for Submission.pptx]

## Slide 1
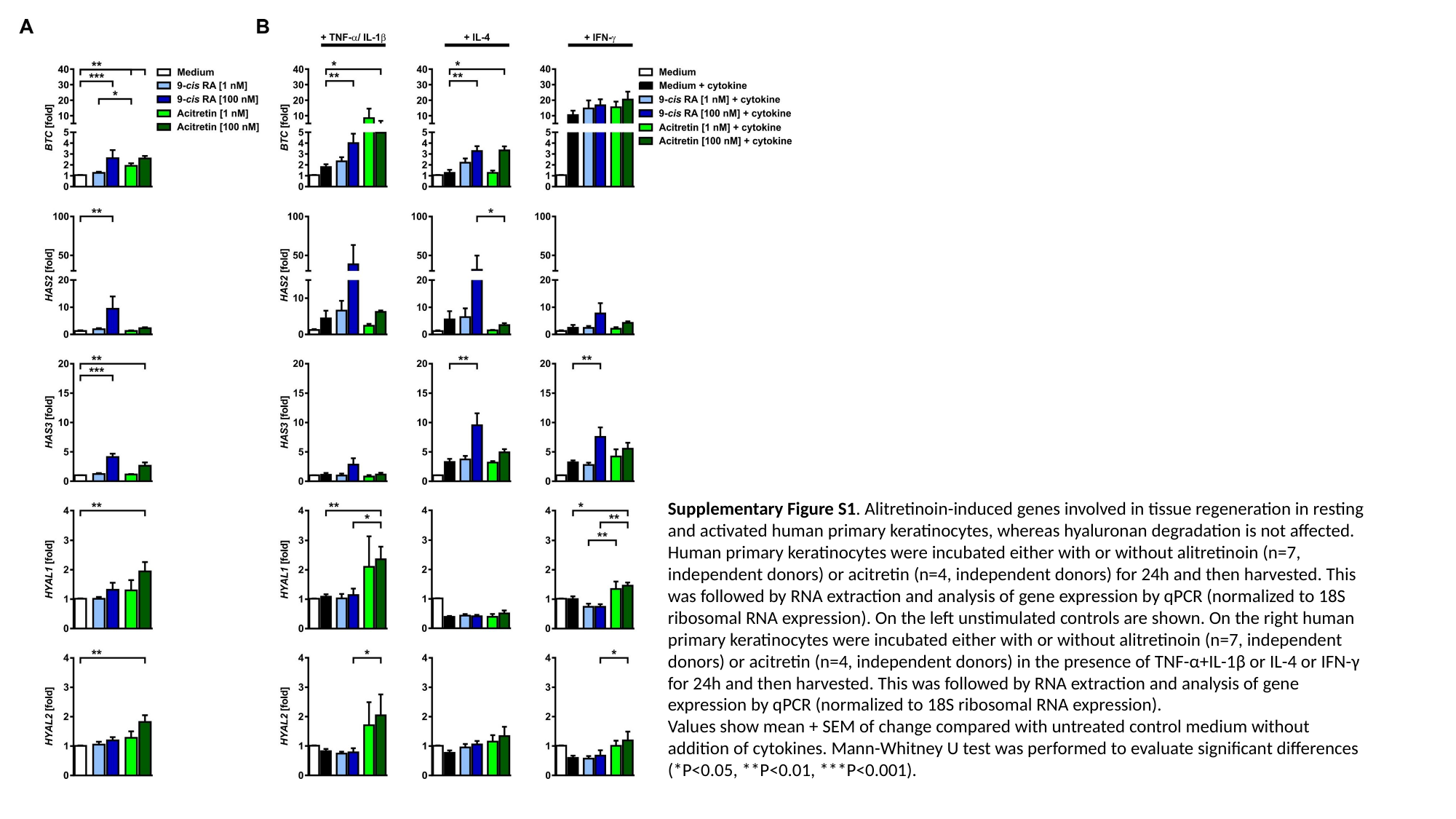

Supplementary Figure S1. Alitretinoin-induced genes involved in tissue regeneration in resting and activated human primary keratinocytes, whereas hyaluronan degradation is not affected.
Human primary keratinocytes were incubated either with or without alitretinoin (n=7, independent donors) or acitretin (n=4, independent donors) for 24h and then harvested. This was followed by RNA extraction and analysis of gene expression by qPCR (normalized to 18S ribosomal RNA expression). On the left unstimulated controls are shown. On the right human primary keratinocytes were incubated either with or without alitretinoin (n=7, independent donors) or acitretin (n=4, independent donors) in the presence of TNF-α+IL-1β or IL-4 or IFN-γ for 24h and then harvested. This was followed by RNA extraction and analysis of gene expression by qPCR (normalized to 18S ribosomal RNA expression).
Values show mean + SEM of change compared with untreated control medium without addition of cytokines. Mann-Whitney U test was performed to evaluate significant differences (*P<0.05, **P<0.01, ***P<0.001).

## Slide 2
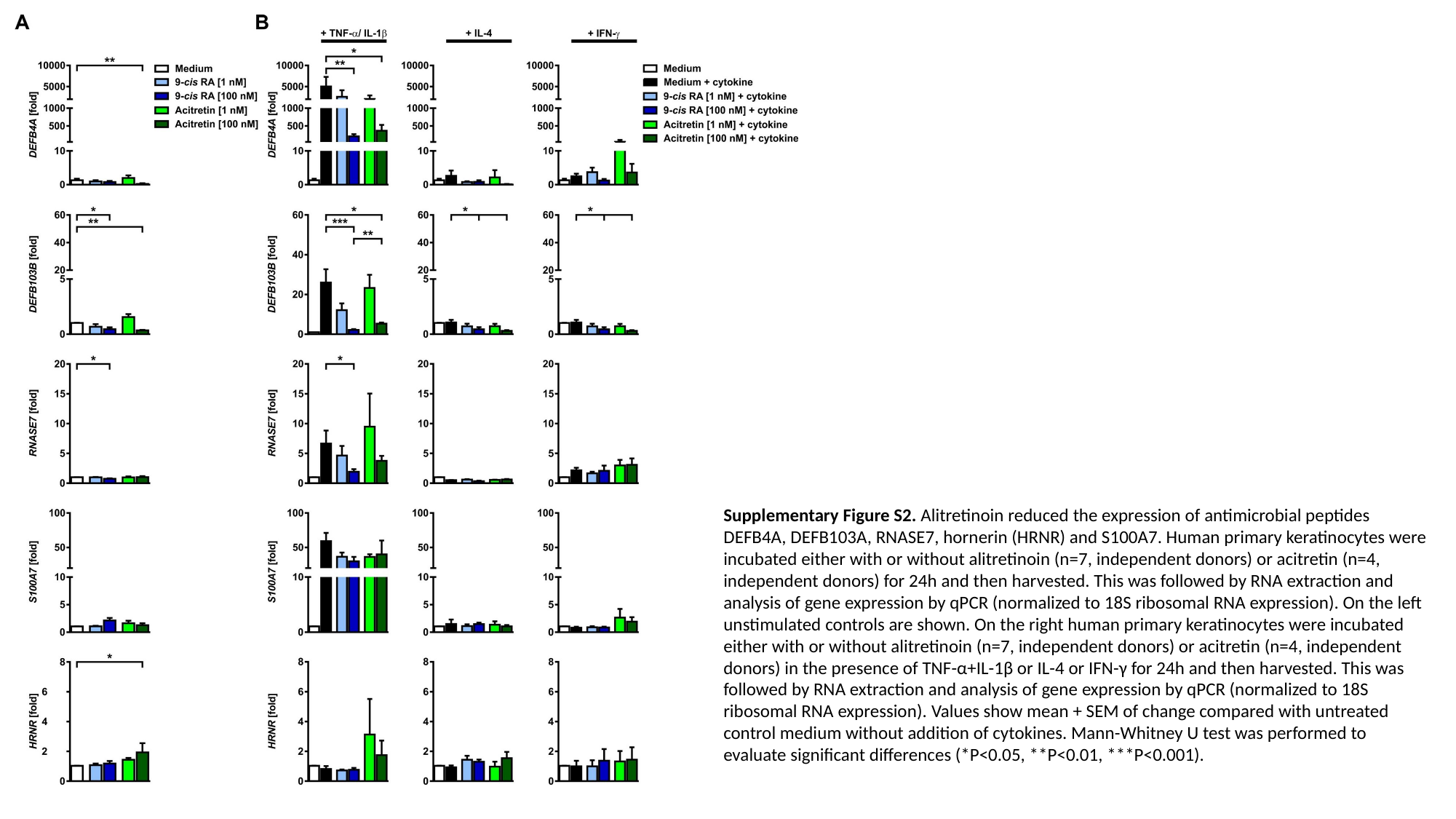

Supplementary Figure S2. Alitretinoin reduced the expression of antimicrobial peptides DEFB4A, DEFB103A, RNASE7, hornerin (HRNR) and S100A7. Human primary keratinocytes were incubated either with or without alitretinoin (n=7, independent donors) or acitretin (n=4, independent donors) for 24h and then harvested. This was followed by RNA extraction and analysis of gene expression by qPCR (normalized to 18S ribosomal RNA expression). On the left unstimulated controls are shown. On the right human primary keratinocytes were incubated either with or without alitretinoin (n=7, independent donors) or acitretin (n=4, independent donors) in the presence of TNF-α+IL-1β or IL-4 or IFN-γ for 24h and then harvested. This was followed by RNA extraction and analysis of gene expression by qPCR (normalized to 18S ribosomal RNA expression). Values show mean + SEM of change compared with untreated control medium without addition of cytokines. Mann-Whitney U test was performed to evaluate significant differences (*P<0.05, **P<0.01, ***P<0.001).

## Slide 3
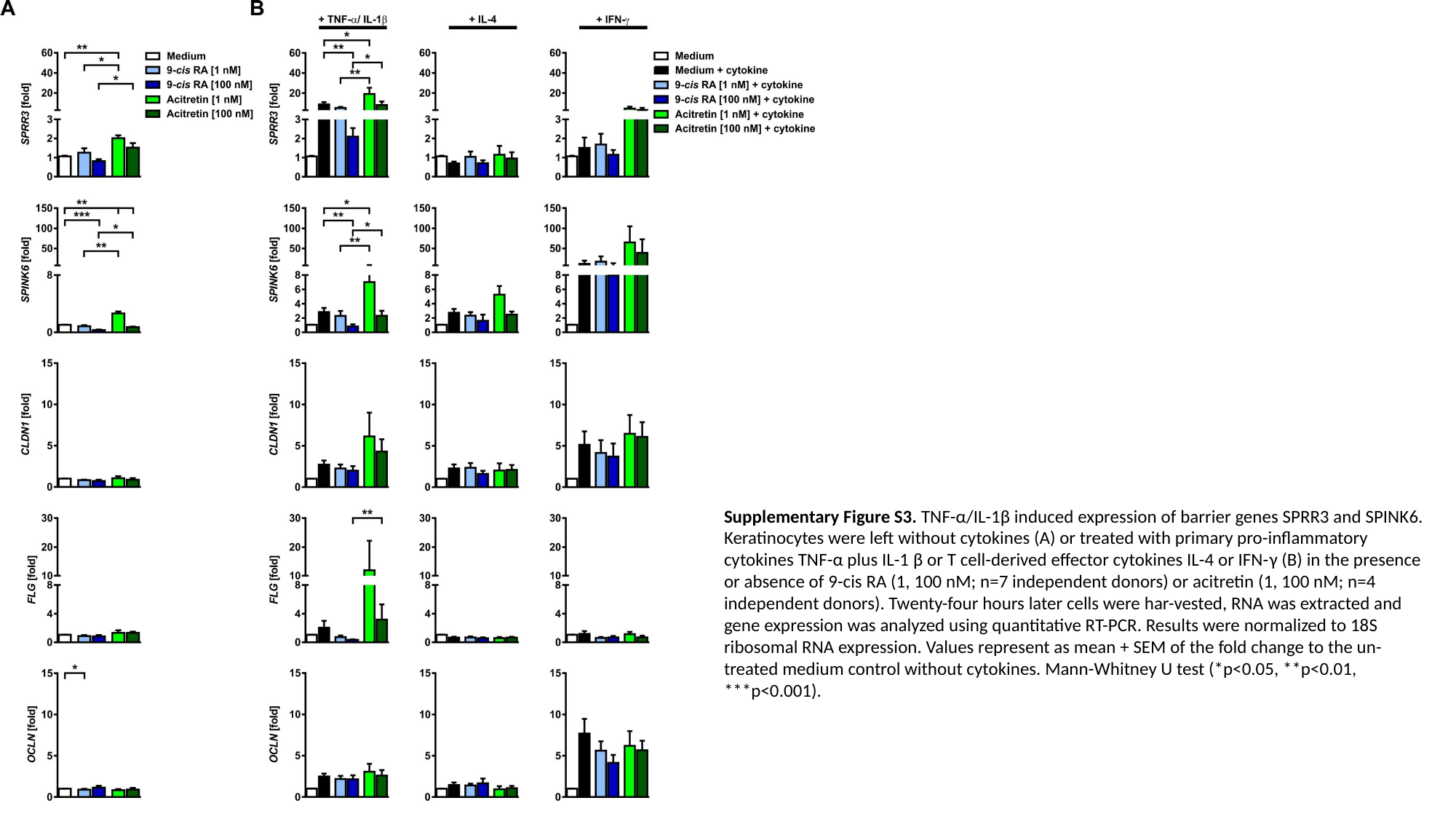

Supplementary Figure S3. TNF-α/IL-1β induced expression of barrier genes SPRR3 and SPINK6. Keratinocytes were left without cytokines (A) or treated with primary pro-inflammatory cytokines TNF-α plus IL-1 β or T cell-derived effector cytokines IL-4 or IFN-γ (B) in the presence or absence of 9-cis RA (1, 100 nM; n=7 independent donors) or acitretin (1, 100 nM; n=4 independent donors). Twenty-four hours later cells were har-vested, RNA was extracted and gene expression was analyzed using quantitative RT-PCR. Results were normalized to 18S ribosomal RNA expression. Values represent as mean + SEM of the fold change to the un-treated medium control without cytokines. Mann-Whitney U test (*p<0.05, **p<0.01, ***p<0.001).

## Slide 4
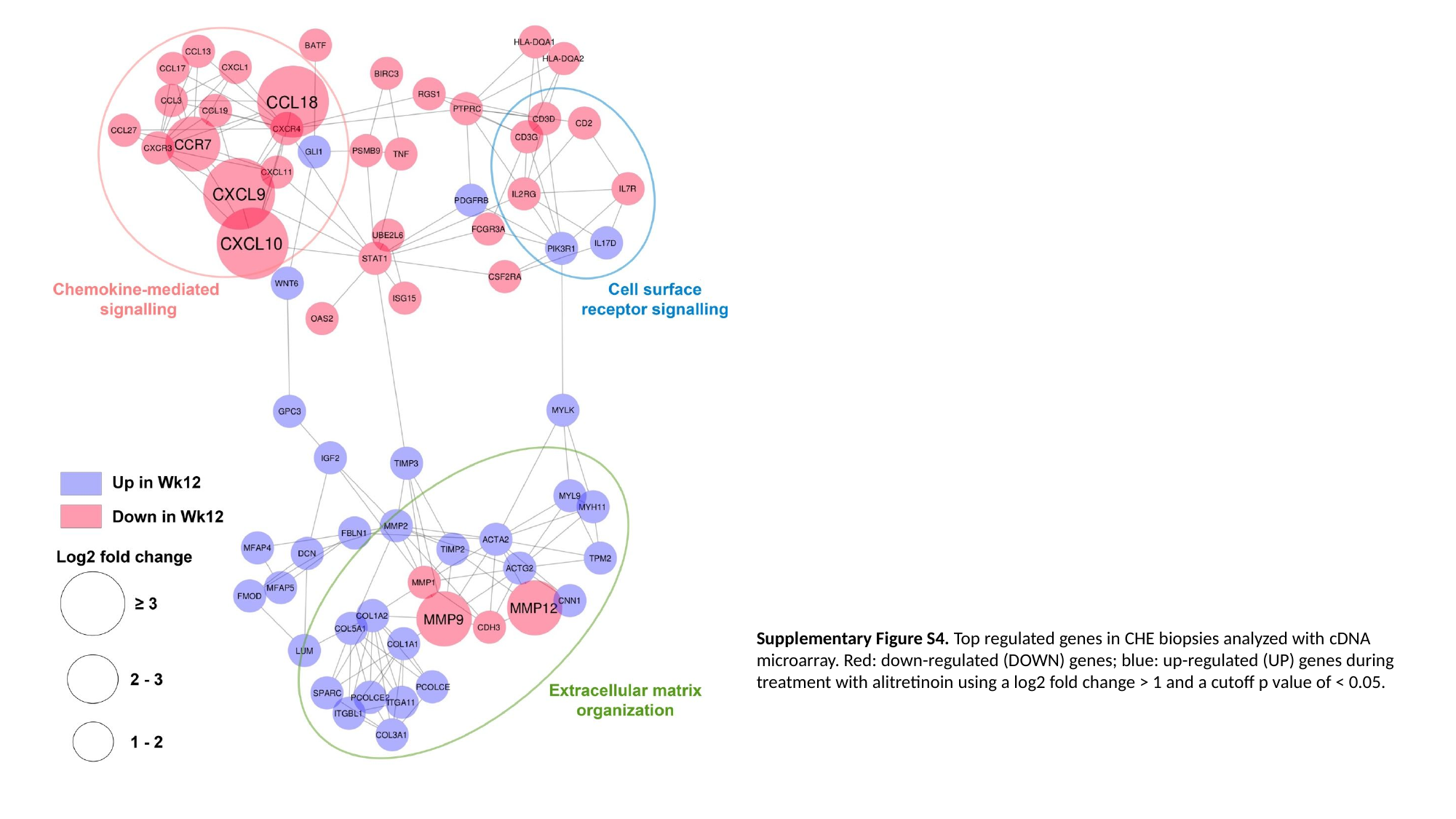

Supplementary Figure S4. Top regulated genes in CHE biopsies analyzed with cDNA microarray. Red: down-regulated (DOWN) genes; blue: up-regulated (UP) genes during treatment with alitretinoin using a log2 fold change > 1 and a cutoff p value of < 0.05.

## Slide 5
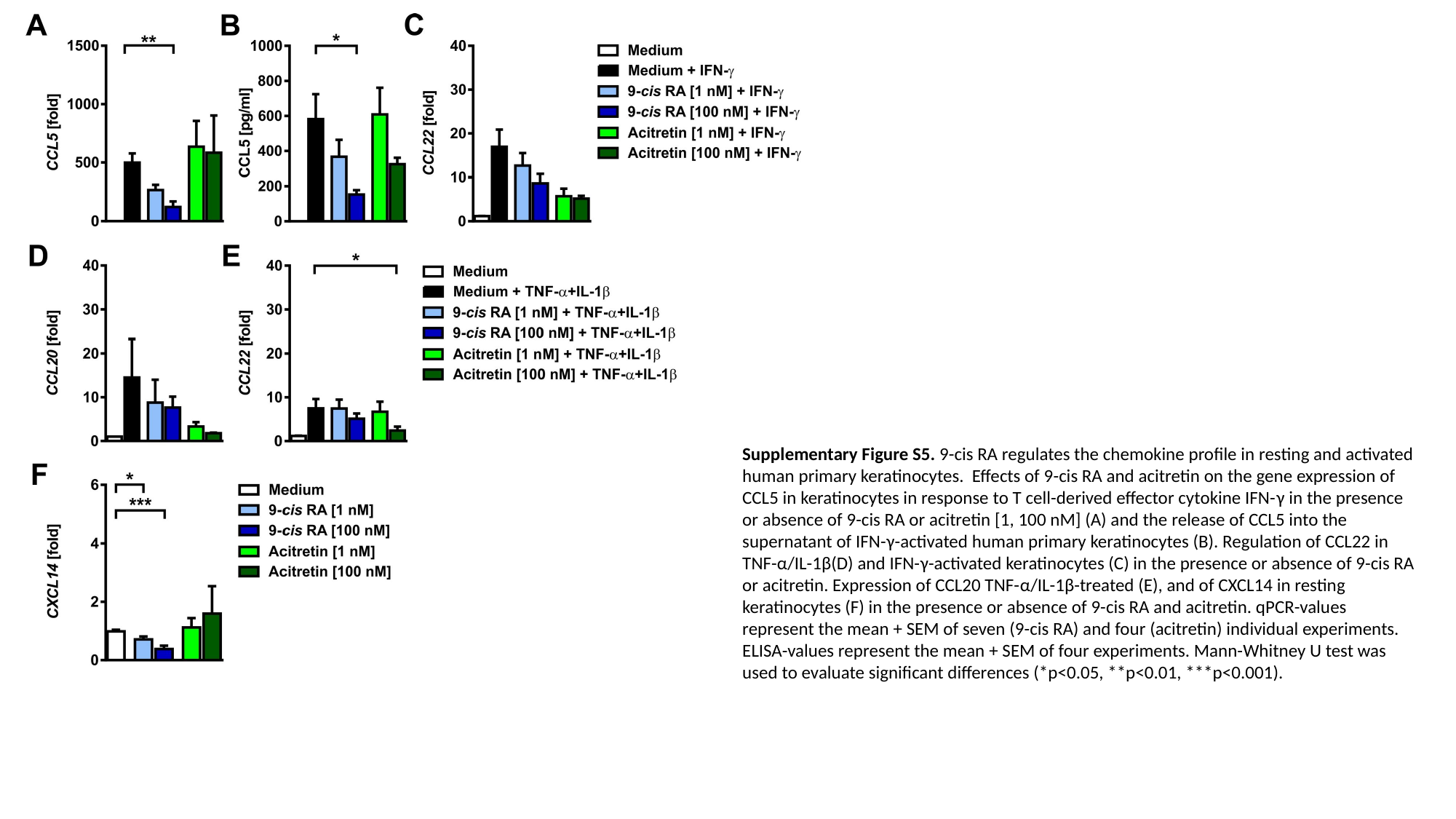

Supplementary Figure S5. 9-cis RA regulates the chemokine profile in resting and activated human primary keratinocytes. Effects of 9-cis RA and acitretin on the gene expression of CCL5 in keratinocytes in response to T cell-derived effector cytokine IFN-γ in the presence or absence of 9-cis RA or acitretin [1, 100 nM] (A) and the release of CCL5 into the supernatant of IFN-γ-activated human primary keratinocytes (B). Regulation of CCL22 in TNF-α/IL-1β(D) and IFN-γ-activated keratinocytes (C) in the presence or absence of 9-cis RA or acitretin. Expression of CCL20 TNF-α/IL-1β-treated (E), and of CXCL14 in resting keratinocytes (F) in the presence or absence of 9-cis RA and acitretin. qPCR-values represent the mean + SEM of seven (9-cis RA) and four (acitretin) individual experiments. ELISA-values represent the mean + SEM of four experiments. Mann-Whitney U test was used to evaluate significant differences (*p<0.05, **p<0.01, ***p<0.001).

## Slide 6
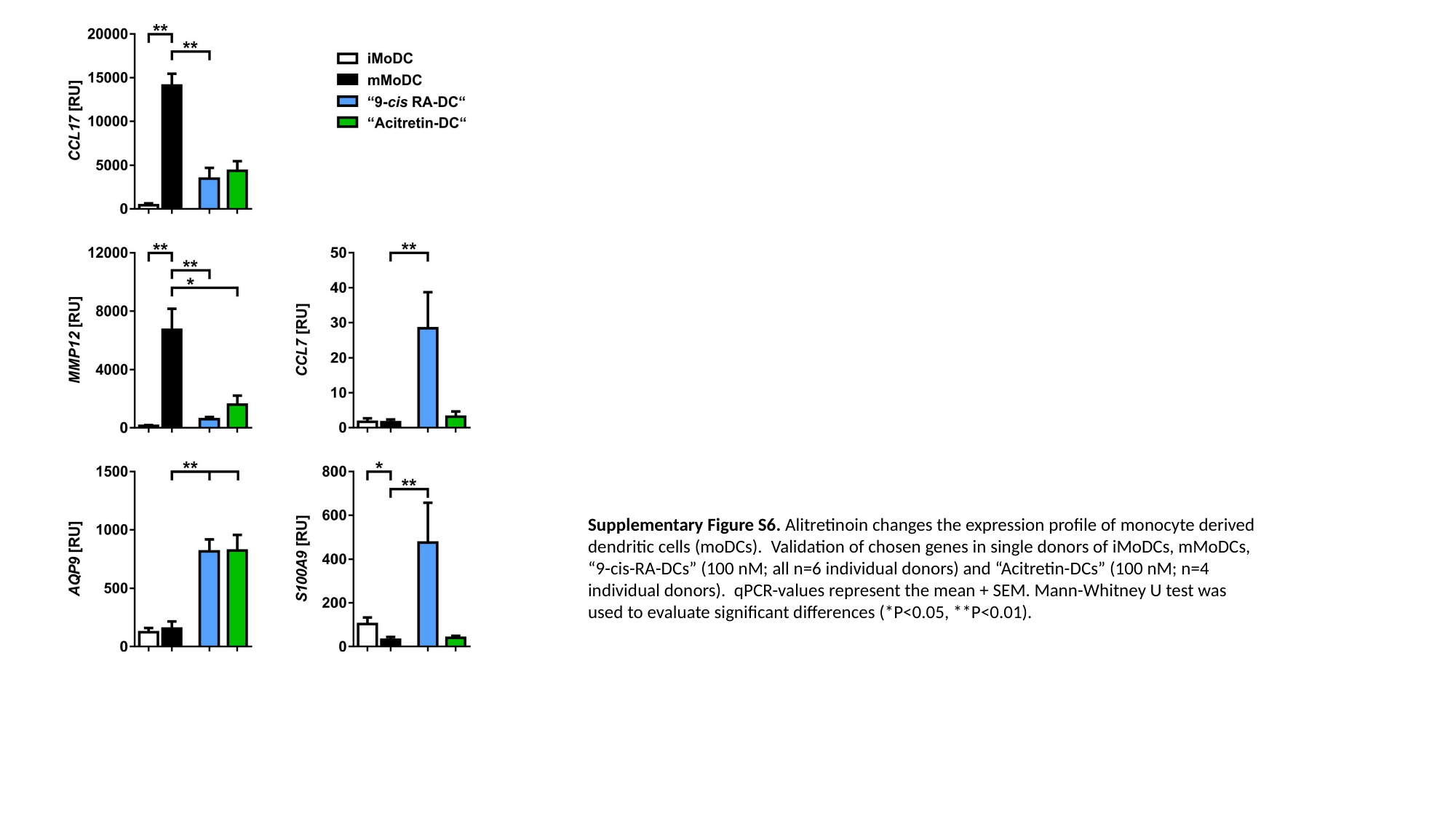

Supplementary Figure S6. Alitretinoin changes the expression profile of monocyte derived dendritic cells (moDCs). Validation of chosen genes in single donors of iMoDCs, mMoDCs, “9-cis-RA-DCs” (100 nM; all n=6 individual donors) and “Acitretin-DCs” (100 nM; n=4 individual donors). qPCR-values represent the mean + SEM. Mann-Whitney U test was used to evaluate significant differences (*P<0.05, **P<0.01).

## Slide 7
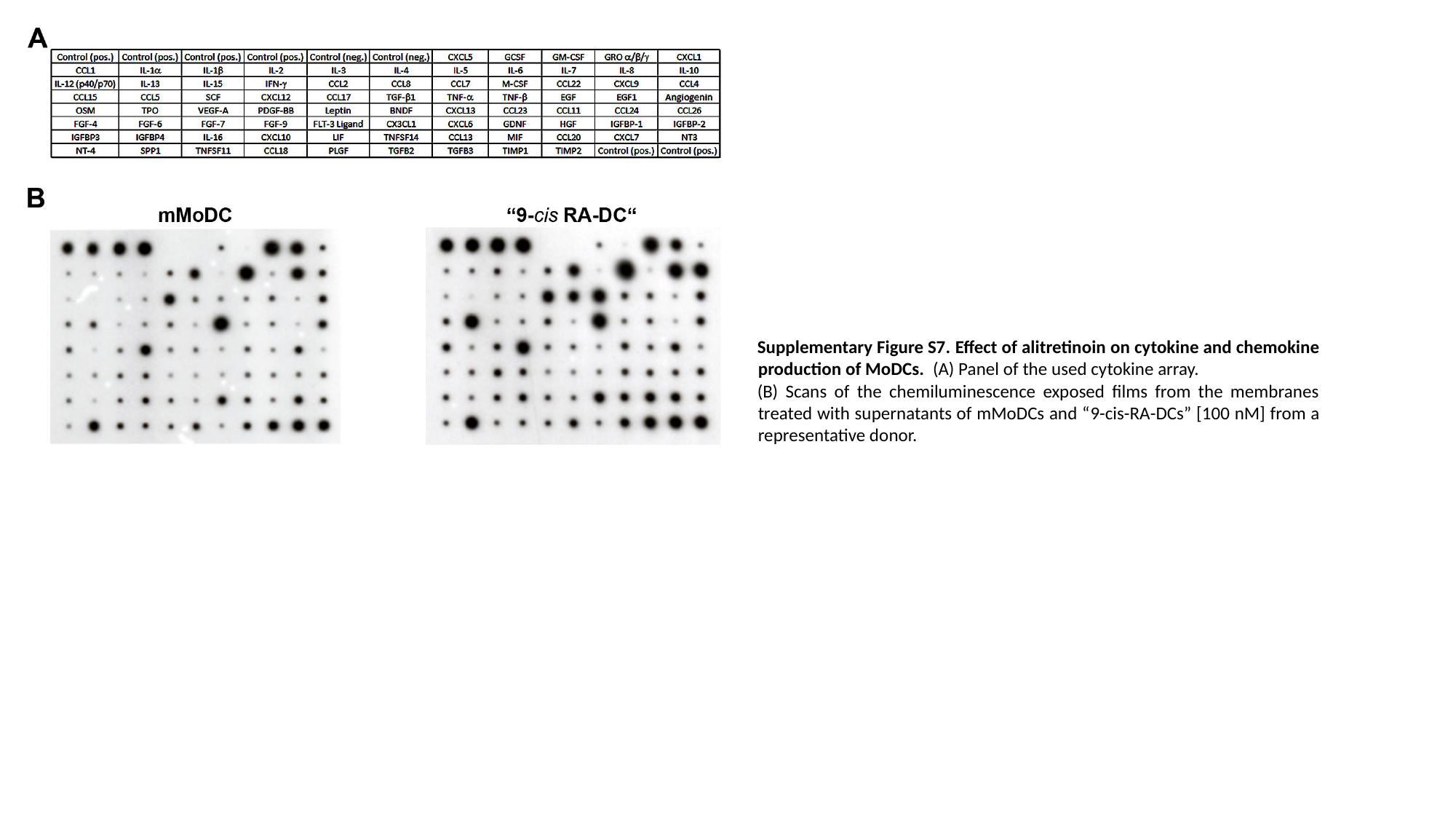

Supplementary Figure S7. Effect of alitretinoin on cytokine and chemokine production of MoDCs. (A) Panel of the used cytokine array.
(B) Scans of the chemiluminescence exposed films from the membranes treated with supernatants of mMoDCs and “9-cis-RA-DCs” [100 nM] from a representative donor.

## Slide 8
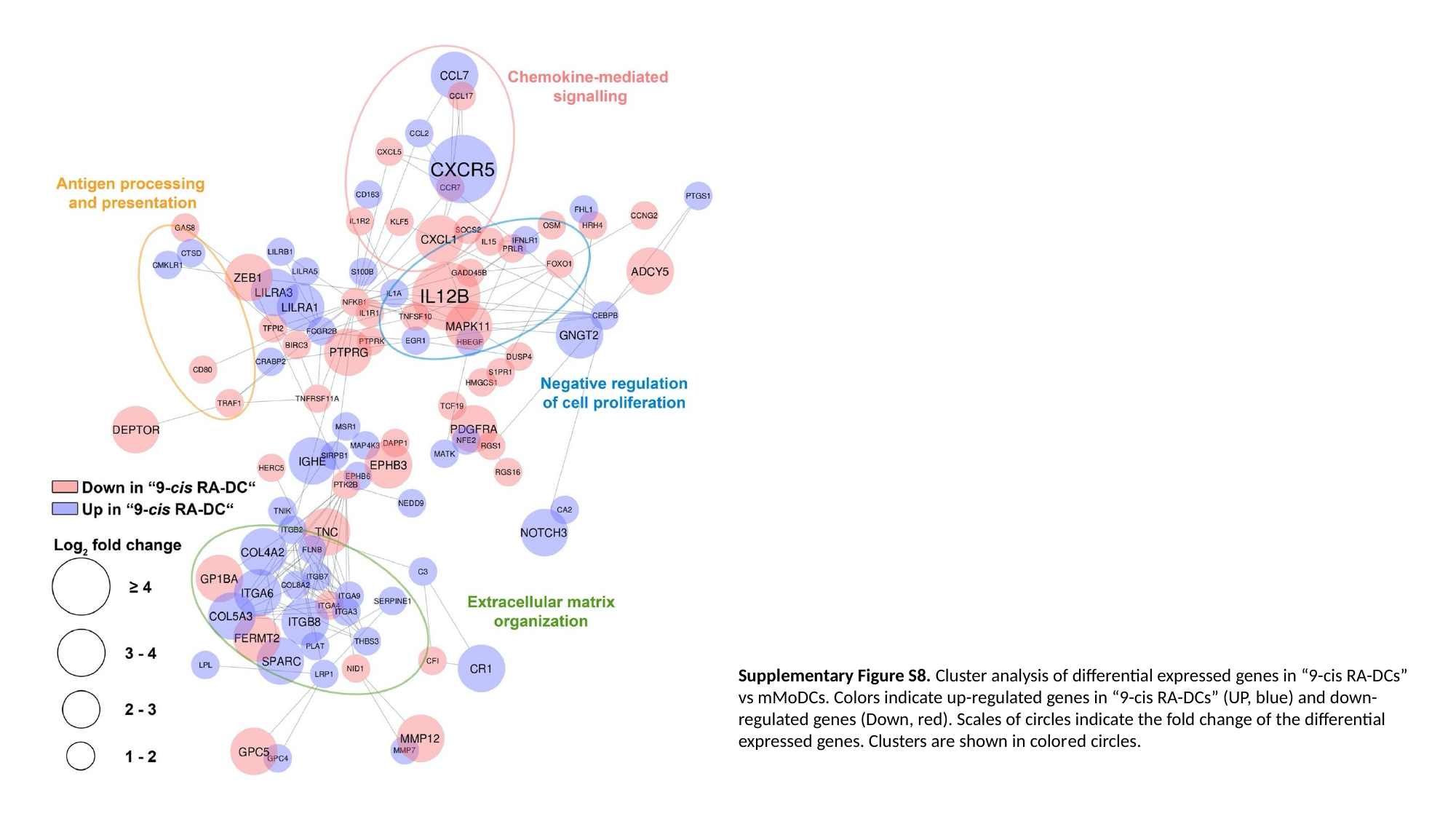

Supplementary Figure S8. Cluster analysis of differential expressed genes in “9-cis RA-DCs” vs mMoDCs. Colors indicate up-regulated genes in “9-cis RA-DCs” (UP, blue) and down-regulated genes (Down, red). Scales of circles indicate the fold change of the differential expressed genes. Clusters are shown in colored circles.

## Slide 9
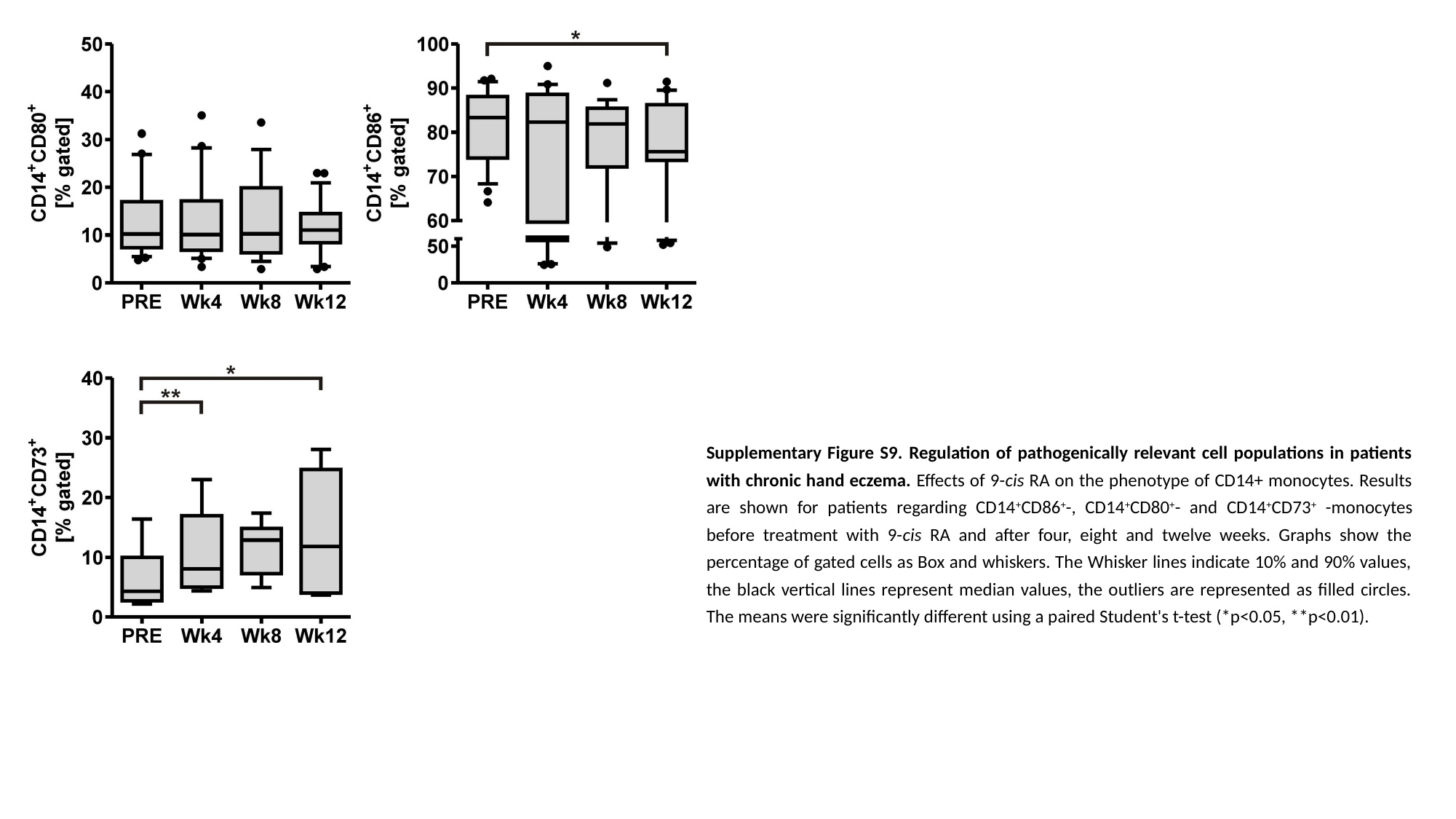

Supplementary Figure S9. Regulation of pathogenically relevant cell populations in patients with chronic hand eczema. Effects of 9-cis RA on the phenotype of CD14+ monocytes. Results are shown for patients regarding CD14+CD86+-, CD14+CD80+- and CD14+CD73+ -monocytes before treatment with 9-cis RA and after four, eight and twelve weeks. Graphs show the percentage of gated cells as Box and whiskers. The Whisker lines indicate 10% and 90% values, the black vertical lines represent median values, the outliers are represented as filled circles. The means were significantly different using a paired Student's t-test (*p<0.05, **p<0.01).
